# Skin Resilience and Biological Adaptability: Current Clinical Evidence on Micronutrients and Bioactive Compounds

**DOI:** 10.3390/biomedicines14092111

**Published:** 2026-09-18

**Authors:** Daniela Mihalache, Alina-Mihaela Gurau, Gabriela Patrichi, Gabriela Gurau, Catalin-Bogdan Satala

**Affiliations:** 1 Faculty of Medicine and Pharmacy, Medical and Pharmaceutical Research Center, “Dunărea de Jos” University of Galati, 800008 Galati, Romania; daniela.mihalache@ugal.ro (D.M.); gabriela.gurau@ugal.ro (G.G.); catalin.satala@ugal.ro (C.-B.S.); 2 Department of Pathology, Clinical County Emergency Hospital Braila, 810325 Braila, Romania; 3 The School for Doctoral Studies in Biomedical Sciences, “Dunărea de Jos” University of Galați, 800008 Galati, Romania; 4 The Doctoral School of Medicine and Pharmacy, “George Emil Palade” University of Medicine, Pharmacy, Science and Technology of Targu Mures, 540142 Targu Mures, Romania

**Keywords:** skin resilience, skin aging, micronutrients, bioactive compounds, carotenoids, antioxidants, photoprotection

## Abstract

Skin resilience and biological adaptability describe the capacity of cutaneous tissue to preserve structural integrity and functional stability under intrinsic aging and environmental stress. Rather than introducing a novel biological pathway, this framework integrates established domains, including redox homeostasis, extracellular matrix maintenance, inflammatory regulation, and barrier function, into a measurable construct. Micronutrients and bioactive compounds participate in these processes; however, the strength and clinical relevance of current evidence require critical evaluation. This structured narrative review appraises mechanistic and clinical studies published between 2000 and 2025 investigating nutritional modulation of measurable skin parameters. Electronic database searches (PubMed/MEDLINE, Scopus, Web of Science) were conducted using predefined terms related to skin aging, resilience-related endpoints, and micronutrient interventions. Controlled human trials were prioritized, and mechanistic findings were distinguished from clinical outcomes. Current clinical evidence most consistently supports a role for carotenoids and antioxidant vitamins in enhancing cutaneous redox stability and short-term photoprotection. Collagen-based formulations and selected polyphenols demonstrate modest improvements in elasticity, hydration, and wrinkle-related parameters, though many trials are limited by small sample sizes, short duration, and heterogeneous endpoints. Overall, micronutrients appear to function primarily as modulators of adaptive capacity rather than as primary drivers of structural rejuvenation. Larger, longer-term randomized trials employing standardized resilience-related endpoints are needed to clarify the magnitude and durability of clinically meaningful effects.

## 1. Introduction

The skin is a dynamic and metabolically active organ that functions as a barrier against environmental stressors while maintaining systemic and local homeostasis [1]. Throughout life, it is continuously exposed to ultraviolet radiation, pollution, oxidative stress, and metabolic fluctuations. The capacity of the skin to withstand these stressors, preserve structural integrity, and maintain functional stability has been increasingly described under the concept of skin resilience [2]. In dermatologic research, skin resilience does not represent a novel biological mechanism but rather an integrative framework encompassing established processes such as redox homeostasis, extracellular matrix (ECM) maintenance, inflammatory regulation, barrier integrity, and cellular adaptive responses [3,4]. Operationally, skin resilience may be reflected by measurable parameters including transepidermal water loss (TEWL), dermal collagen density, skin elasticity, wrinkle depth, antioxidant capacity, and inflammatory responses [5,6,7,8]. These parameters provide quantifiable boundaries that allow resilience to be evaluated in both experimental and clinical settings. Aging, particularly photoaging, represents a progressive decline in cutaneous adaptive capacity [7,9,10]. Hallmarks include increased oxidative burden, impaired ECM remodeling, mitochondrial dysfunction, and chronic low-grade inflammation (“inflammaging”) [11,12,13]. These interconnected processes gradually narrow the functional range within which the skin can respond proportionately to environmental challenges. Preserving adaptive range rather than merely preventing visible aging signs has emerged as a central concept in contemporary dermatologic research. Micronutrients and bioactive compounds, including carotenoids, vitamins C and E, trace elements such as zinc and selenium, and selected phytochemicals, participate in multiple pathways that underpin skin resilience [14,15]. They contribute to antioxidant defense systems, modulate inflammatory signaling, influence collagen synthesis, and interact with transcriptional networks regulating cellular stress responses. While their biological roles are well described individually, the strength, consistency, and translational relevance of current evidence remain heterogeneous. Existing reviews have addressed nutritional influences on skin aging, antioxidant protection, or specific micronutrients. However, few have critically appraised the extent to which available preclinical and clinical data collectively support the concept of nutritional modulation of skin resilience as an integrative outcome. A structured evaluation distinguishing levels of evidence, translational applicability, and methodological limitations is therefore warranted. This review aims to critically appraise current evidence regarding micronutrients and bioactive compounds as modulators of skin resilience in aging. By differentiating mechanistic data from clinical outcomes and evaluating the quality and limitations of existing studies, we seek to clarify the degree to which nutritional interventions may meaningfully influence measurable parameters of cutaneous adaptability.

## 2. Literature Search Strategy and Study Selection

### 2.1. Search Strategy

This review was conducted as a structured narrative synthesis designed to critically appraise the current evidence regarding micronutrients and bioactive compounds as potential modulators of skin resilience and biological adaptability. The review was not designed as a formal systematic review or meta-analysis; however, methodological elements of the PRISMA 2020 framework were applied to improve transparency in the identification, screening, eligibility assessment, and reporting of the retrieved literature.

Two reviewers independently screened the records identified in PubMed/MEDLINE, Scopus, and Web of Science, first at the title and abstract level and subsequently at the full-text level. Disagreements regarding eligibility were resolved through discussion and consensus between the reviewers. The search covered publications from 2000 to 2025 and was designed around three conceptual domains: (1) skin resilience, skin aging, photoaging, and related cutaneous adaptive processes; (2) micronutrients and bioactive compounds; and (3) measurable clinical, structural, biochemical, and functional skin outcomes. Search terms within and across these conceptual domains were combined using Boolean operators, with database-specific adaptations made according to the indexing characteristics of each database and the use of controlled vocabulary where applicable. The complete database-specific search strings are provided in Supplementary Table S1.

The search strategy was intentionally broad because the concept of skin resilience encompasses multiple interconnected biological domains rather than a single standardized clinical endpoint. Accordingly, terms relating to epidermal barrier function, transepidermal water loss (TEWL), hydration, elasticity, collagen and dermal structure, wrinkles, erythema, pigmentation, oxidative stress, antioxidant activity, inflammatory responses, and photoprotection were considered alongside intervention-related terms.

Reference lists of relevant primary studies and review articles were additionally screened manually to identify potentially eligible publications not retrieved through the electronic database searches.

**Figure 1:**
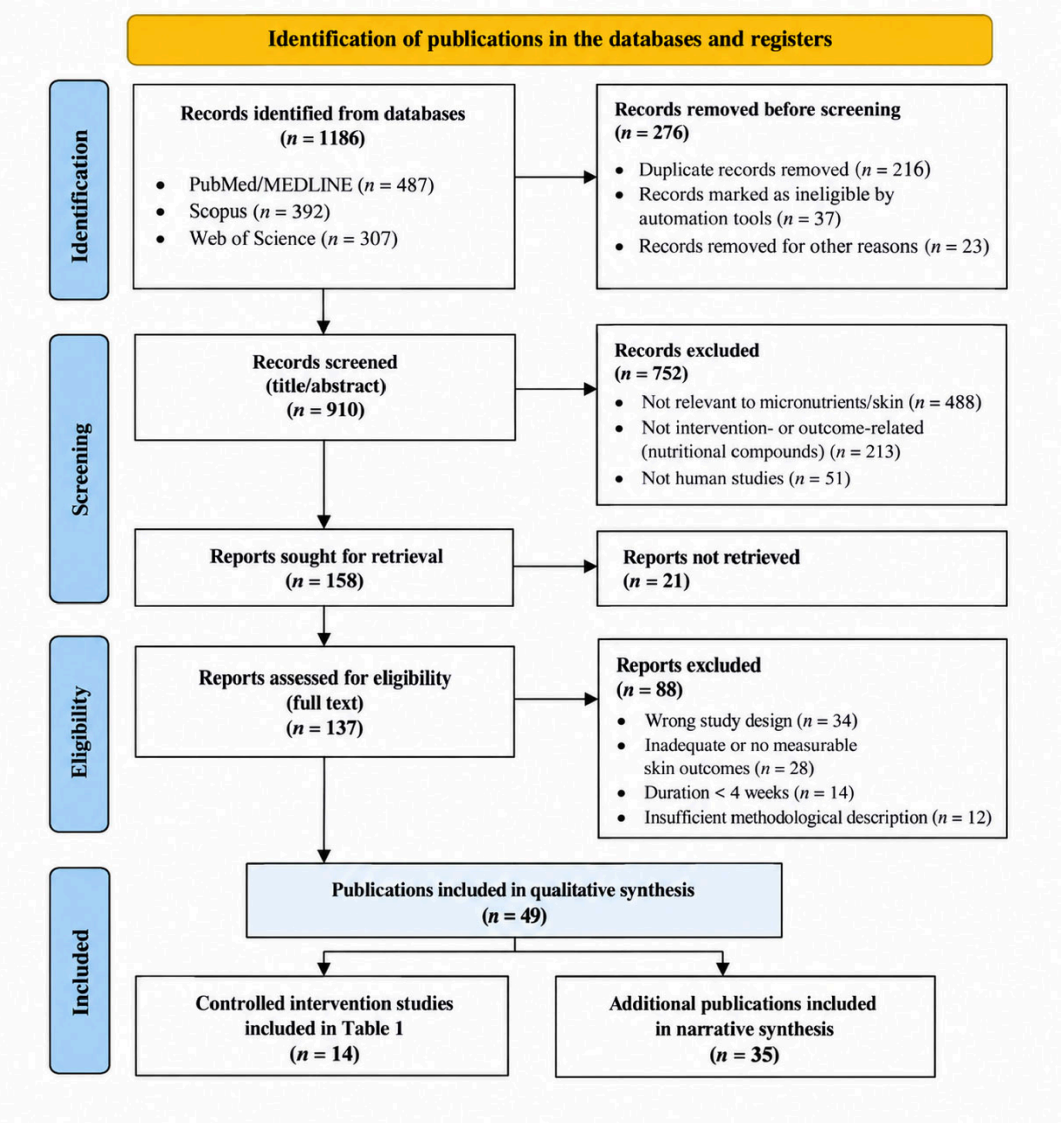
Flow diagram illustrating the literature search, study selection, eligibility assessment, and inclusion process used for this structured narrative review. The diagram summarizes the identification, screening, and selection of studies according to predefined eligibility criteria.

The electronic database search identified 1186 records: 487 from PubMed/MEDLINE, 392 from Scopus, and 307 from Web of Science. After removal of duplicate records and records excluded before formal screening, 910 records remained for screening. Following title and abstract assessment, 154 reports were sought for retrieval and 137 full-text reports were assessed for eligibility. Ultimately, 49 publications were included in the qualitative synthesis. Of these, 14 controlled human intervention studies were considered sufficiently relevant, while the remaining 35 publications contributed complementary clinical, observational, mechanistic, or contextual evidence to the narrative synthesis. The complete set of additional publications is provided in Supplementary Table S2, while the study-selection pathway is presented in Figure 1.

### 2.2. Eligibility Criteria

Studies were considered eligible when they investigated micronutrients or bioactive compounds administered orally or topically and reported measurable skin-related outcomes relevant to skin resilience, biological adaptability, aging, photoaging, barrier function, structural integrity, oxidative balance, inflammatory regulation, or responses to environmental stress.

Eligible evidence comprised randomized controlled trials, non-randomized or controlled intervention studies, observational studies, skin-specific mechanistic investigations, and systematic or narrative reviews. Human clinical studies were given priority when evaluating clinical effects. Randomized and controlled intervention studies were considered the principal source of evidence for intervention efficacy, whereas observational studies were used primarily to provide contextual or associative evidence, mechanistic studies to support biological plausibility, and review articles to provide conceptual context and to identify relevant primary studies.

The intervention category included vitamins, carotenoids, minerals, polyphenols and other plant-derived compounds, antioxidant compounds, and other bioactive substances investigated in relation to measurable cutaneous outcomes. Collagen peptides and collagen-containing formulations were considered separately from conventional micronutrient interventions because collagen represents a protein-derived bioactive substrate rather than a micronutrient. Combined formulations were retained when their composition and clinical outcomes were sufficiently described, but their multicomponent nature was considered when interpreting efficacy and comparability across studies.

Oral and topical interventions were both eligible because the review addresses nutritional and bioactive modulation of skin resilience across different routes of exposure. However, route of administration was considered an important source of biological heterogeneity. Topical pharmacologically active compounds, particularly topical retinoids such as retinol, were therefore distinguished from conventional oral nutritional supplementation and were not interpreted as equivalent nutritional interventions.

Particular attention was given to baseline nutritional status. Where information was available, studies were interpreted according to whether participants had documented or presumed nutritional insufficiency, adequate baseline nutritional status, or no reported assessment of baseline micronutrient status. This distinction was considered important because supplementation in individuals with inadequate intake or deficiency may have different biological effects from supplementation in nutritionally sufficient populations.

Studies were excluded when they focused exclusively on wound healing without relevance to aging or adaptive cutaneous responses; investigated systemic outcomes without measurable dermatologic endpoints; were editorials, conference abstracts, or case reports without sufficient primary data; or lacked adequate methodological information for interpretation. Studies in which the investigated outcome could not reasonably be related to one or more measurable dimensions of skin resilience were also excluded.

The principal skin outcomes considered relevant to the resilience framework included TEWL, epidermal hydration, elasticity and firmness, dermal or collagen density, wrinkle depth or severity, erythema and pigmentation responses, antioxidant capacity, oxidative-stress markers, inflammatory biomarkers, and other objectively measurable structural or functional cutaneous parameters. Static skin characteristics were interpreted as correlates of resilience rather than as direct measures of resilience unless the study incorporated an environmental, oxidative, inflammatory, or other relevant stressor and assessed the capacity to maintain or recover function.

Because eligible evidence encompassed different study designs and intervention categories, evidence was not pooled quantitatively. Instead, findings were weighted according to study design, methodological strength, directness of the measured endpoint to the resilience framework, intervention characteristics, duration of follow-up, and consistency across independent studies. Primary studies incorporated within systematic or narrative reviews were not counted as independent additional evidence when the same findings were already represented in the primary-study synthesis; reviews were used primarily for contextual interpretation and identification of relevant literature.

### 2.3. Data Extraction and Evidence Synthesis

For each included study, information relevant to the review question was extracted and organized according to study design, population characteristics, intervention characteristics, route of administration, duration of exposure, measured skin outcomes, principal findings, and major methodological considerations. For controlled human intervention studies, the extracted information included sample size, participant characteristics, intervention composition and dose where reported, comparator, route of administration, intervention duration, primary or principal skin endpoints, direction of the reported effect, and relevant methodological limitations. Particular attention was given to whether outcomes were reported as between-group differences, changes from baseline, or within-group changes, because these approaches are not equivalent when interpreting intervention efficacy.

The evidence was synthesized narratively rather than quantitatively because of substantial heterogeneity in interventions, formulations, doses, routes of administration, populations, outcome definitions, measurement techniques, and follow-up periods. Particular consideration was given to whether an observed outcome represented a direct functional measure of cutaneous adaptation or a surrogate correlate of resilience. Thus, improvements in hydration, elasticity, wrinkle parameters, erythema, or biochemical antioxidant markers were not automatically interpreted as evidence of enhanced long-term skin resilience. Controlled human intervention studies were summarized separately to provide a transparent overview of the principal clinical evidence. The remaining eligible literature was incorporated into the narrative synthesis according to its evidentiary role, with mechanistic and preclinical findings used to explain biological plausibility rather than to establish clinical efficacy.

### 2.4. Assessment of Study Quality and Risk Bias

The methodological strength of the available evidence was considered in the interpretation of the findings, with particular attention to study design, sample size, intervention duration, endpoint selection, measurement methodology, and the presence of an appropriate comparator.

For controlled intervention studies, the principal methodological domains considered were randomization, allocation and blinding where applicable, adequacy of the control group, completeness of outcome assessment, attrition, selective outcome reporting, and potential conflicts of interest or industry funding. Particular caution was applied to small studies, multicomponent formulations, short intervention periods, surrogate endpoints, and studies in which baseline nutritional status was not characterized.

Observational studies were interpreted with attention to population selection, exposure assessment, potential confounding, and the distinction between associations and intervention effects. Mechanistic and preclinical studies were assessed primarily according to the directness of their findings to human cutaneous biology and were not considered equivalent in evidentiary weight to controlled human trials.

Because the included literature was methodologically heterogeneous and the review was structured as a narrative rather than a formal systematic review with meta-analysis, evidence was not reduced to a single pooled estimate of effect. Instead, the methodological limitations of individual studies were incorporated into the qualitative weighting of the evidence and explicitly considered when drawing conclusions regarding clinical relevance and durability.

## 3. Operational Framework of Skin Resilience

To critically evaluate the contribution of micronutrients and bioactive compounds to skin resilience, the concept must first be translated into an operational framework that distinguishes resilience from conventional descriptions of skin aging, photoaging, or individual skin characteristics. In this review, resilience is therefore used as an integrative analytical construct rather than as a distinct biological mechanism. It provides a framework for considering whether nutritional or bioactive interventions may help preserve cutaneous function and adaptive capacity across interconnected biological domains.

### 3.1. Conceptual and Operational Definition

In dermatologic research, skin resilience can be understood as the capacity of cutaneous tissue to preserve functional and structural stability while responding to intrinsic aging and repeated environmental or metabolic stress. It therefore overlaps with established concepts of skin homeostasis, aging, and photodamage but is not synonymous with any of them [2,7,10,11].

The added value of the resilience framework is primarily analytical. Conventional descriptions of skin aging generally focus on the accumulation of structural and molecular changes, such as collagen loss, elastin fragmentation, oxidative damage, barrier impairment, or wrinkle formation. By contrast, resilience emphasizes the capacity of the tissue to maintain an adequate functional range when exposed to stress and, where measurable, to limit or recover from stress-induced disturbance. Thus, resilience shifts the interpretation from the presence of an aging-related feature alone toward the preservation of functional stability across interconnected biological domains.

For the purposes of this review, skin resilience is operationally defined as the coordinated preservation of epidermal barrier competence, dermal structural integrity, redox homeostasis, and inflammatory regulation during aging and in the presence of relevant environmental or metabolic stressors. This definition does not imply resistance to normal aging or reversal of established structural changes. Rather, it describes the extent to which cutaneous systems remain functionally stable and capable of an appropriate adaptive response within physiological limits.

This distinction is important when interpreting clinical endpoints. Parameters such as transepidermal water loss (TEWL), hydration, elasticity, wrinkle depth, erythema, or dermal density are measurable characteristics of the skin, but none of them, considered in isolation, constitutes a direct measure of resilience. They may instead represent structural, biochemical, or functional correlates of resilience. Their interpretation as evidence of enhanced resilience is stronger when the study evaluates maintenance of function during a relevant stressor, attenuation of stress-induced deterioration, or recovery toward baseline following a defined challenge. Conversely, an isolated improvement in a static parameter should not automatically be interpreted as evidence that the adaptive capacity of the skin has been enhanced.

This framework also provides a basis for distinguishing nutritional maintenance from pharmacological rejuvenation. Nutritional adequacy may contribute to preservation of physiological cutaneous function, particularly when baseline nutritional status is suboptimal, but this does not imply reversal of intrinsic aging or substantial reconstruction of aged tissue. The resilience framework therefore emphasizes maintenance, stabilization, and adaptive capacity rather than structural rejuvenation [16,17].

### 3.2. Measurable Dimensions of Cutaneous Resilience

The multidimensional nature of skin resilience requires evaluation across complementary structural, functional, biochemical, and clinical domains. No single endpoint is sufficient to characterize resilience; rather, convergent changes across several domains provide a more appropriate basis for interpretation.

Barrier function represents one of the most directly measurable dimensions of cutaneous functional stability. TEWL provides an objective measure of water loss across the epidermal barrier, while stratum corneum hydration and, where assessed, epidermal lipid organization provide complementary information regarding barrier condition [6,18]. Stable or appropriately regulated TEWL in the presence of environmental challenge may support preservation of barrier homeostasis, whereas persistent elevation may indicate impaired barrier function. However, changes in hydration or TEWL measured under resting conditions should be interpreted primarily as changes in barrier-related skin characteristics unless an intervention study demonstrates preservation or recovery of barrier function following a defined stressor.

Dermal structural integrity and mechanical function constitute a second major dimension. Collagen density, dermal density, elastin organization, elasticity, firmness, and measures of matrix turnover provide complementary information regarding the structural state of the dermis [19,20,21,22]. Matrix metalloproteinase activity and other markers of extracellular matrix degradation may provide biochemical correlates of structural deterioration. Improvements in these parameters may indicate favorable effects on dermal structure or mechanical properties; however, short-term changes should not be equated automatically with modification of the long-term trajectory of cutaneous aging.

Redox homeostasis represents a further dimension of resilience because cutaneous tissues are continuously exposed to endogenous and environmental sources of oxidative stress [23,24]. Cutaneous carotenoid concentrations, antioxidant enzyme activity, total antioxidant capacity, and biomarkers of oxidative damage can provide measurable indicators of the redox environment [14]. Evidence that an intervention reduces oxidative disturbance or preserves antioxidant capacity under an appropriate challenge is more directly relevant to resilience than an isolated increase in an antioxidant biomarker without evidence of functional consequences.

Inflammatory regulation is another component of adaptive capacity. Persistent low-grade inflammatory signaling has been associated with cutaneous aging and may interact with oxidative stress and extracellular matrix degradation [11,12]. Tissue or circulating inflammatory mediators, cytokine profiles, and other validated inflammatory biomarkers may therefore provide complementary evidence regarding inflammatory regulation. As with other domains, changes in inflammatory biomarkers should be interpreted cautiously and should not be considered direct evidence of enhanced resilience unless they are linked to preservation or recovery of cutaneous function.

Clinical responses to environmental stress provide an additional and particularly relevant dimension. UV-induced erythema, photoprotective responses, changes in pigmentation, and other experimentally induced responses can provide information about how skin responds to an external challenge. These endpoints may therefore have greater conceptual relevance to resilience than static measures obtained without a stressor. Nevertheless, attenuation of an acute erythematous response should be interpreted as evidence of short-term photobiologic modulation rather than as proof of long-term prevention of skin aging.

Wrinkle severity, surface roughness, pigmentation, hydration, and elasticity remain clinically relevant outcomes because they reflect the cumulative consequences of structural and functional processes occurring in aging skin. Within the present framework, however, they are treated primarily as resilience-associated phenotypes rather than direct measures of resilience. The evidentiary interpretation therefore depends on the context in which these endpoints were measured, the duration of follow-up, the presence or absence of an environmental challenge, and whether the observed change was demonstrated relative to an appropriate control.

Collectively, these domains provide a multidimensional framework in which resilience is inferred from the preservation or modulation of interconnected cutaneous functions rather than from a single anti-aging endpoint. This approach also allows clinical findings to be distinguished from mechanistic plausibility and prevents improvements in isolated surrogate parameters from being interpreted as definitive evidence of enhanced long-term skin resilience.

### 3.3. Biological Adaptability and the Aging Trajectory

Closely related to resilience is the concept of biological adaptability, which refers to the dynamic capacity of skin cells, particularly keratinocytes and fibroblasts, to mount proportionate responses to environmental and metabolic stress [25]. Adaptive processes include activation of antioxidant pathways, regulation of inflammatory cascades, maintenance of mitochondrial function, and controlled extracellular matrix remodeling [7,26]. With advancing age, this adaptive range progressively narrows. Mitochondrial inefficiency, accumulated oxidative damage, altered proteostasis, and persistent inflammatory signaling collectively reduce the capacity of cutaneous tissue to respond effectively to stress [26]. Clinically, this decline manifests as increased TEWL, reduced elasticity, impaired dermal density, and heightened sensitivity to environmental exposure. From a nutritional standpoint, micronutrients and bioactive compounds may influence adaptability by supporting enzymatic antioxidant systems, modulating transcriptional responses to stress, contributing to collagen biosynthesis, and regulating inflammatory mediators. Within this framework, nutritional interventions are not positioned as anti-aging cures but as potential modulators of adaptive range [26]. Importantly, this operational model does not propose novel biological pathways. Rather, it integrates established mechanisms into a measurable construct that enables critical evaluation of current evidence. By defining resilience and adaptability through quantifiable domains, subsequent sections of this review will assess whether available mechanistic and clinical data substantiate meaningful nutritional modulation of these parameters.

**Figure 2:**
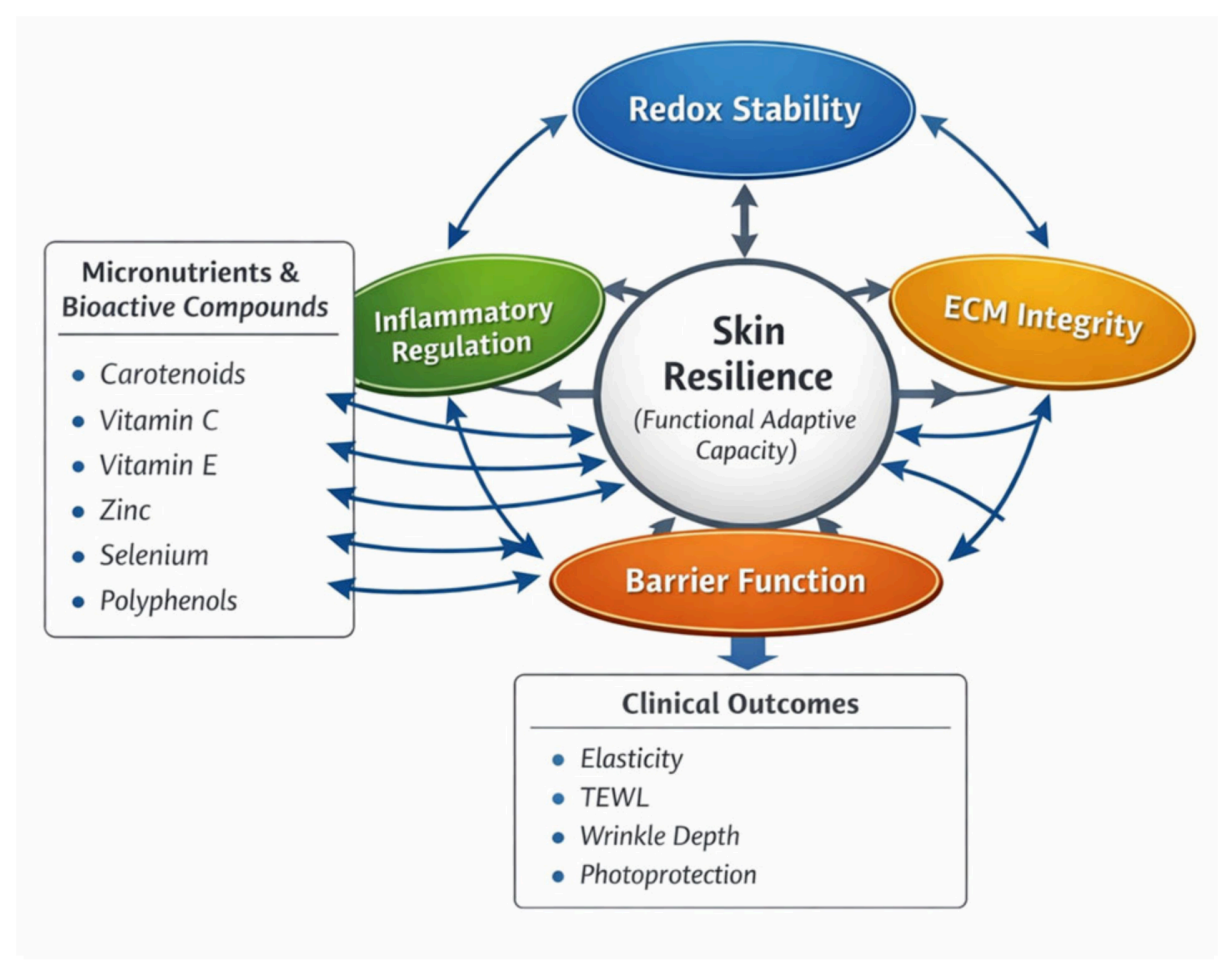
Multidimensional model of skin resilience and point of nutritional modulation.

Figure 2 schematically summarizes the multidimensional structure of skin resilience and highlights the principal biological domains through which micronutrients and bioactive compounds may exert modulatory effects.

## 4. Nutritional Modulation of Core Domains of Skin Resilience

### 4.1. Redox Stability as a Primary Target of Nutritional Modulation

Oxidative imbalance represents one of the most consistently implicated drivers of cutaneous aging and functional decline. Both intrinsic metabolic activity and extrinsic factors, particularly ultraviolet radiation, generate reactive oxygen species that influence keratinocyte turnover, fibroblast function, and extracellular matrix remodeling. The biological plausibility that antioxidant micronutrients may support skin resilience is therefore strong. The key question, however, is not whether antioxidants can neutralize reactive species in experimental systems, but whether nutritional interventions meaningfully influence measurable parameters of redox balance and downstream structural outcomes in humans.

Carotenoids are among the most extensively investigated compounds in this regard. These lipophilic pigments accumulate within the epidermis and contribute to the cutaneous antioxidant network [27,28]. Controlled human studies demonstrate that oral carotenoid supplementation increases skin carotenoid levels and enhances resistance to UV-induced erythema [28,29]. Such findings provide evidence of short-term photobiological protection. However, most intervention trials are of relatively short duration and rely primarily on acute photobiological endpoints. Data linking carotenoid supplementation to long-term modulation of dermal matrix preservation or visible wrinkle progression remain limited and methodologically heterogeneous [14].

Vitamin C occupies a central position within the antioxidant network and collagen biosynthesis pathways. In vitro data clearly demonstrate that adequate ascorbate availability enhances collagen production in fibroblasts and reduces oxidative stress markers. Observational human studies have associated higher dietary vitamin C intake with improved skin appearance metrics. Nevertheless, controlled supplementation trials specifically evaluating isolated oral vitamin C effects on aging skin parameters are fewer in number and vary in design. Improvements in oxidative biomarkers are more consistently reported than durable structural changes [30].

Vitamin E functions primarily as a lipid-phase antioxidant, protecting cellular membranes and the stratum corneum from peroxidative damage. Evidence for its efficacy is strongest in topical applications or when administered in combination with other antioxidants. Oral supplementation studies show variable results, suggesting that synergistic interactions within antioxidant networks may be more relevant than single-agent approaches. Trace elements such as selenium and zinc contribute indirectly to redox control by supporting enzymatic antioxidant systems. While mechanistic rationale is robust, clinical trials specifically examining their isolated impact on aging-related skin outcomes remain relatively sparse. Much of the available evidence derives from systemic nutritional research rather than dermatology-focused trials [31,32].

This evidence base supports the capacity of selected micronutrients to enhance cutaneous antioxidant status and improve short-term photoprotection. However, the magnitude, durability, and clinical significance of structural anti-aging effects remain less clearly established. Distinguishing between enhancement of antioxidant biomarkers, short-term photobiological protection, and meaningful modification of aging trajectories is therefore essential when interpreting claims regarding improved skin resilience [33].

### 4.2. Extracellular Matrix Preservation and Structural Integrity

While redox stability represents a proximal defense mechanism, preservation of extracellular matrix (ECM) architecture defines the structural core of skin resilience. Age-related dermal alterations are characterized by reduced collagen synthesis, increased matrix metalloproteinase (MMP) activity, elastin fragmentation, and altered glycosaminoglycan distribution. These changes collectively impair mechanical properties and contribute to visible aging features such as reduced elasticity and wrinkle formation. The translational relevance of nutritional modulation in this domain therefore depends on whether micronutrients meaningfully influence collagen dynamics and matrix remodeling in vivo [34,35].

Vitamin C provides the strongest mechanistic rationale for ECM support, given its indispensable role as a cofactor in collagen hydroxylation. In vitro and ex vivo studies consistently demonstrate enhanced collagen production in fibroblasts under adequate ascorbate availability. However, clinical translation is more nuanced. Oral supplementation trials frequently report modest improvements in skin density or elasticity parameters, yet these effects are often observed in small cohorts and over limited follow-up periods. Moreover, baseline nutritional status is rarely stratified, making it difficult to distinguish corrective effects in individuals with inadequate intake or nutritional status from effects in already sufficient populations [30,36,37,38].

Carotenoids may indirectly influence matrix stability by attenuating oxidative signaling pathways that stimulate MMP expression. Experimental models show reduced MMP-1 activation following antioxidant exposure, suggesting potential preservation of collagen integrity. In human trials, reductions in UV-induced MMP expression have been documented, but evidence demonstrating sustained increases in dermal collagen density or long-term structural remodeling remains limited. Thus, while suppression of degradative pathways is biologically plausible, robust confirmation of durable ECM preservation through nutritional intervention is still emerging.

Polyphenols and other plant-derived bioactives have attracted attention for their capacity to modulate signaling cascades involved in matrix turnover. Preclinical data suggest downregulation of collagen-degrading enzymes and support for fibroblast function. Yet, human evidence is heterogeneous, often relying on composite antioxidant formulations that make attribution to specific compounds difficult.

Overall, nutritional support of ECM integrity is mechanistically credible and supported by short-term intervention data. However, the magnitude of clinically meaningful structural change remains modest compared with procedural or pharmacologic dermatologic interventions. Nutritional modulation may therefore be best interpreted as a supportive factor contributing to maintenance of structural stability rather than as a primary driver of matrix regeneration [39,40,41,42,43,44].

### 4.3. Modulation of Inflammatory Tone and Inflammaging

Chronic low-grade inflammation has emerged as a defining feature of biological aging, including within the skin. Persistent activation of pro-inflammatory signaling pathways contributes to matrix degradation, impaired cellular repair, and amplification of oxidative stress [11,45,46]. Within the resilience framework, effective regulation of inflammatory tone is essential for preserving adaptive capacity.

Micronutrients influence inflammatory pathways through several mechanisms, including modulation of redox-sensitive transcription factors and cytokine production. Carotenoids and polyphenols have demonstrated capacity to attenuate NF-κB activation and reduce expression of pro-inflammatory mediators in experimental systems [39,40]. Vitamins C and E may indirectly dampen inflammatory cascades by limiting oxidative amplification of cytokine signaling. Human evidence in the context of aging skin, however, is less abundant than mechanistic data. Many clinical studies focus on acute photoinflammatory responses, such as erythema intensity following UV exposure, rather than chronic inflammatory markers associated with aging [35].

Reductions in UV-induced erythema provide evidence of anti-inflammatory and photoprotective activity, yet they do not necessarily equate to sustained modification of inflammaging processes. Observational research suggests that higher dietary antioxidant intake correlates with improved skin appearance and reduced dryness, but these associations are subject to confounding variables, including overall lifestyle factors.

Consequently, although biological plausibility for inflammatory modulation is strong, direct evidence linking micronutrient supplementation to long-term reduction in age-associated cutaneous inflammatory burden remains limited. Nutritional interventions may therefore contribute to maintaining inflammatory balance, particularly under environmental stress, but their role in reversing established inflammaging appears modest based on currently available data [11,35].

### 4.4. Barrier Function and Epidermal Adaptability

The epidermal barrier represents the most immediate interface between environmental exposure and systemic physiology. Maintenance of barrier integrity is central to skin resilience, as impaired permeability control increases susceptibility to oxidative stress, microbial imbalance, and chronic inflammation. Nutritional factors may influence barrier competence through effects on keratinocyte differentiation, lipid synthesis, and antioxidant defense within the stratum corneum. Carotenoids accumulate preferentially in superficial epidermal layers, contributing to localized antioxidant protection. Vitamin E stabilizes lipid membranes, while zinc supports keratinocyte proliferation and enzymatic activity involved in epidermal renewal. Clinical trials evaluating oral antioxidant supplementation frequently report modest improvements in TEWL and hydration parameters, particularly in individuals with low baseline antioxidant levels. However, methodological variability—including differences in measurement devices, environmental controls, and population characteristics—complicates cross-study comparison. Moreover, improvements in hydration or resting TEWL do not necessarily demonstrate restoration of adaptive barrier capacity or fundamental alteration of epidermal lipid architecture [38,43,47,48].

Evidence supporting barrier enhancement through nutrition is therefore suggestive but variable in magnitude. Nutritional adequacy likely contributes to maintaining epidermal stability, yet robust data demonstrating substantial restoration of impaired barrier function solely through supplementation are limited [38,43,47,48].

### 4.5. Integrative Perspective Across Domains

When considered collectively, the evidence suggests that micronutrients and bioactive compounds most consistently influence redox stability and acute photoprotection. Effects on extracellular matrix preservation, chronic inflammatory modulation, and barrier restoration are biologically plausible but supported by more heterogeneous and often short-term clinical data. Importantly, the magnitude of observed changes is typically modest. Nutritional modulation appears to operate within physiological boundaries, supporting maintenance of adaptive capacity rather than inducing dramatic structural transformation. This interpretation aligns with the resilience framework proposed earlier: micronutrients may help preserve functional range, particularly under environmental stress, but current evidence does not support the conclusion that supplementation alone substantially alters the intrinsic trajectory of cutaneous aging.

A critical appraisal of study quality further underscores this perspective. Many intervention trials involve small sample sizes, limited follow-up duration, and surrogate endpoints. Few large-scale, long-term randomized controlled trials have evaluated hard structural outcomes such as dermal thickness progression or wrinkle evolution over multiple years. Thus, while the biological rationale for nutritional modulation of skin resilience is well grounded, the strength of clinical evidence varies across domains and compounds. Clear differentiation between mechanistic plausibility, surrogate changes, and demonstrated clinical magnitude is essential for balanced interpretation [35,47,49].

### 4.6. Clinical Evidence from Controlled Trials

To complement the mechanistic and experimental evidence discussed above, we identified controlled intervention studies published between 2000 and 2025 that evaluated defined micronutrient or bioactive compound interventions in relation to measurable skin parameters. These clinical trials are summarized in Table 1. Their findings are interpreted according to study design, intervention characteristics, duration, comparator, and the nature of the measured skin endpoint rather than being considered equivalent evidence of skin resilience. Additional publications contributing to the qualitative synthesis are summarized in Supplementary Table S2.

Overall, these trials are characterized by relatively small sample sizes, short intervention periods (typically 8–16 weeks), and frequent use of combined formulations. Most studies report improvements in selected skin parameters, but heterogeneity in endpoints, formulations, and study populations limits direct comparability across interventions [35,36,37,38,42,43,44,47,48,49,53].

## 5. Oral Versus Topical Nutritional Strategies: Bioavailability and Cutaneous Relevance

The clinical interpretation of bioactive interventions requires distinction between oral and topical administration. Although both routes may influence measurable skin parameters, their biological effects depend on systemic bioavailability, tissue distribution, local penetration, and cutaneous metabolism [44,51].

### 5.1. Oral Supplementation and Systemic Delivery

Oral administration is the predominant route evaluated for micronutrients and dietary bioactives. Following absorption, compounds reach the skin through systemic circulation, although cutaneous availability varies substantially between nutrients. Carotenoids provide a well-documented example, as oral supplementation increases measurable skin carotenoid levels and may enhance resistance to UV-induced erythema [35,47,52,54].

The magnitude of the cutaneous response is influenced by baseline nutritional status and systemic metabolism. Increasing oral intake does not necessarily produce proportional increases in tissue bioactivity. This may partly explain the modest effects observed in some supplementation trials, particularly in nutritionally replete populations [36,38,54].

Collagen peptides represent a distinct category of oral bioactive intervention and should not be classified as micronutrients. Clinical trials of collagen-containing formulations have reported improvements in parameters such as dermal density, collagen structure, elasticity, and wrinkle measures, although many formulations also contain vitamin C or other bioactive ingredients [37,38,48]. Their effects should therefore be interpreted separately from those of conventional micronutrient supplementation.

### 5.2. Topical Delivery and Localized Effects

Topical application bypasses systemic distribution and delivers active compounds directly to epidermal and superficial dermal compartments. This route may achieve higher local concentrations than oral supplementation, particularly for lipophilic antioxidants such as vitamin E or certain carotenoids. Topical antioxidants have demonstrated efficacy in reducing acute UV-induced oxidative damage and improving photoprotection. However, penetration depth is influenced by molecular size, lipophilicity, formulation, and stratum corneum integrity. Furthermore, topical application may predominantly affect epidermal processes, with more limited impact on deeper dermal structures where collagen remodeling occurs. Importantly, the biological effects of topically applied compounds cannot be assumed to replicate their systemic metabolic roles. For example, topical vitamin C may influence local collagen synthesis, but its stability and cutaneous penetration depend strongly on formulation chemistry [55,56,57,58].

Topical retinol should be considered separately from conventional nutritional interventions. In dermatologic formulations, retinol acts as a pharmacologically active topical retinoid rather than as a nutritional supplement. The clinical evidence from Kafi et al. therefore provides evidence for a topical pharmacological intervention and should not be interpreted as evidence for oral micronutrient supplementation [51].

### 5.3. Comparative and Synergistic Considerations

Direct comparisons between oral and topical strategies are limited. In practice, combined approaches may offer complementary effects: systemic supplementation may support overall nutritional and antioxidant status, whereas topical formulations may provide targeted local effects. Nevertheless, the available literature rarely stratifies outcomes according to baseline nutritional status, degree of photoexposure, or concurrent dermatologic treatments. As a result, extrapolation to clinical practice requires caution. Overall, both oral and topical interventions can influence selected skin parameters, particularly those related to oxidative balance and acute photoprotection. However, differences in bioavailability, tissue distribution, formulation, and study design limit direct comparison of their effects on skin resilience [44,47,52].

### 5.4. Safety, Tolerability and Clinical Considerations

Although the nutritional and bioactive interventions discussed in this review are generally associated with favourable short-term tolerability, safety remains dependent on dose, duration, baseline nutritional status, and concomitant therapies. Excessive intake should not be assumed to provide additional cutaneous benefit, particularly for fat-soluble antioxidants and trace elements. Vitamin E, selenium, and other antioxidant micronutrients exert dose-dependent biological effects, and supplementation beyond nutritional requirements may increase the potential for adverse effects or clinically relevant interactions [31,32].

Safety considerations are particularly important in susceptible populations, including individuals receiving medications that affect coagulation, pregnant women, and patients using multiple supplements simultaneously. Multicomponent formulations may further complicate safety assessment because both efficacy and adverse effects cannot readily be attributed to individual ingredients. Moreover, the predominantly short duration of available clinical trials limits conclusions regarding long-term exposure.

Topical bioactive compounds should be considered separately from oral nutritional supplementation. Pharmacologically active topical retinoids, in particular, may cause local irritation and should not be interpreted as equivalent to conventional nutritional interventions. Overall, current evidence supports cautious, physiologically oriented supplementation aimed at nutritional adequacy rather than supraphysiological exposure [30,31,32,54].

## 6. Inter-Individual Variability and Determinants of Nutritional Responsiveness

The effects of micronutrient supplementation on skin resilience are unlikely to be uniform across individuals. Variability in metabolic status, environmental exposure, age, and baseline nutritional sufficiency may substantially influence responsiveness to nutritional interventions. Baseline antioxidant status represents a particularly important determinant. Individuals with suboptimal dietary intake or increased oxidative burden, such as smokers or those with high cumulative ultraviolet exposure, may derive greater measurable benefit from supplementation than nutritionally replete populations. Many clinical trials, however, do not stratify participants according to baseline micronutrient levels, limiting interpretation of differential response patterns.

Genetic and metabolic differences may also contribute to variability in response, although evidence directly linking specific genetic variants to responsiveness to nutritional interventions in dermatologic contexts remains limited. Age-related metabolic changes further complicate interpretation. Absorption efficiency, tissue distribution, and mitochondrial function evolve across the lifespan, potentially modifying the cutaneous impact of systemic supplementation. Additionally, hormonal status, particularly in postmenopausal populations, may influence extracellular matrix dynamics and inflammatory tone.

Environmental context is another critical variable. Photodamage severity, pollution exposure, and lifestyle factors interact with nutritional status in shaping oxidative and inflammatory load. Consequently, nutritional modulation should be viewed within a multifactorial framework rather than as an isolated determinant. Recognition of inter-individual variability underscores the need for future trials incorporating baseline nutritional assessment, stratified analysis, and personalized approaches to supplementation strategies [54,59].

## 7. Metabolic and Systems-Level Context of Skin Resilience

Skin resilience does not operate independently of systemic metabolic state. Cutaneous cells are influenced by circulating nutrients, endocrine signals, mitochondrial efficiency, and overall metabolic flexibility. Therefore, the impact of micronutrients on skin aging parameters must be interpreted within a broader physiological context.

Mitochondrial function plays a central role in redox regulation and cellular energy availability. Age-associated mitochondrial inefficiency contributes to increased oxidative burden and impaired repair capacity. Certain micronutrients, including antioxidants and trace elements, participate indirectly in supporting mitochondrial enzyme systems, yet direct clinical evidence linking supplementation to improved mitochondrial performance in aging skin remains limited.

Systemic inflammatory tone also influences cutaneous homeostasis. Chronic metabolic conditions characterized by low-grade inflammation may amplify skin aging processes [60,61,62]. Nutritional modulation may exert indirect benefits through systemic anti-inflammatory effects, though isolating cutaneous-specific outcomes remains challenging.

Circadian regulation and metabolic timing represent additional emerging factors. Skin cells exhibit intrinsic circadian rhythms that coordinate DNA repair, barrier renewal, and oxidative defense. Although mechanistic links between micronutrient availability and circadian regulation have been suggested in broader metabolic research, dermatologic-specific evidence is still developing. At present, such interactions should be interpreted as biologically plausible but not yet clinically substantiated.

Collectively, these considerations highlight that nutritional modulation of skin resilience likely reflects integration within a systemic network of metabolic and environmental influences. The relative contribution of isolated supplementation must therefore be contextualized within this broader physiological framework [63].

## 8. Quality of Evidence and Methodological Considerations

Although the biological rationale for nutritional modulation of skin resilience is well established, the strength and consistency of clinical evidence vary substantially across compounds and outcome domains. A critical appraisal of methodological aspects is therefore essential to contextualize reported findings and avoid overinterpretation.

### 8.1. Study Design and Statistical Power

A recurring limitation in this field is the predominance of small to moderately sized clinical trials. Many randomized studies evaluating micronutrient supplementation in aging skin enrol fewer than 60 participants, limiting statistical power and generalizability. While statistically significant improvements in selected parameters are frequently reported, small sample sizes increase vulnerability to both type I and type II errors. Intervention duration further constrains interpretation. Most studies extend between 8 and 16 weeks, a timeframe sufficient to detect changes in antioxidant biomarkers or acute photoprotective responses but arguably insufficient to assess sustained extracellular matrix remodeling or long-term wrinkle progression. Structural dermal changes evolve over months to years; thus, short-term trials may underestimate or misrepresent long-term impact [41].

### 8.2. Endpoint Selection and Measurement Variability

Considerable heterogeneity exists in outcome selection. Studies variably assess transepidermal water loss, hydration, elasticity, erythema response, wrinkle depth, imaging-based dermal density, or biochemical oxidative markers. Differences in measurement devices, environmental controls, and operator technique introduce additional variability. Moreover, reliance on surrogate endpoints, such as reduction in UV-induced erythema, may not directly reflect durable modulation of aging processes. While these measures provide meaningful mechanistic insight, they should not be conflated with definitive evidence of structural rejuvenation or long-term resilience enhancement. Greater standardization of endpoints, incorporation of validated imaging technologies, and longer follow-up intervals would substantially strengthen future investigations [41].

### 8.3. Baseline Nutritional Status and Population Characteristics

Few trials stratify participants according to baseline micronutrient levels. Given homeostatic regulation of many antioxidant systems, supplementation effects are likely more pronounced in individuals with suboptimal status. Failure to account for baseline variability may contribute to inconsistent or modest effect sizes observed across studies. Population heterogeneity further complicates interpretation. Differences in age distribution, cumulative ultraviolet exposure, smoking status, hormonal profile, and comorbid metabolic conditions may influence both baseline resilience and responsiveness to intervention. Stratified analyses remain relatively uncommon in the current literature [64,65].

### 8.4. Translational Interpretation and Clinical Magnitude

A critical distinction must be maintained between statistical significance and clinical relevance. Many reported improvements, particularly in hydration, erythema reduction, or elasticity indices, are modest in absolute magnitude. While these changes may be biologically meaningful, they do not necessarily equate to substantial alteration of visible aging trajectories. Furthermore, extrapolation from in vitro and animal data to human dermatologic outcomes should be approached cautiously. Experimental models often employ concentrations or exposure conditions not directly replicable in physiological settings. Overall, current evidence supports a contributory role for micronutrients in maintaining parameters associated with skin resilience. However, the magnitude of clinical impact appears moderate, and definitive long-term structural modification remains incompletely demonstrated [41].

## 9. Integrative Perspective on Nutritional Support of Skin Resilience

Skin resilience emerges from the coordinated interaction of redox stability, extracellular matrix preservation, inflammatory regulation, and barrier integrity. Disruption in any one domain can amplify vulnerability in others, creating a self-reinforcing cycle of oxidative stress, inflammatory signaling, and structural degradation. Within this interconnected network, micronutrients and bioactive compounds appear to exert modulatory effects primarily by supporting physiological antioxidant systems and maintaining biochemical equilibrium. Their influence is most consistently demonstrated in the context of photoprotection and short-term oxidative stabilization. Effects on long-term matrix preservation, chronic inflammatory modulation, and sustained barrier restoration are biologically plausible but supported by more heterogeneous and often limited-duration clinical data [41]. The resilience framework proposed in this review does not introduce novel biological pathways but rather integrates established dermatologic mechanisms into a multidimensional model suitable for evaluating nutritional interventions. Within this model, nutritional adequacy is best conceptualized as a stabilizing factor that may help preserve adaptive capacity under environmental and metabolic stress, rather than as a primary driver of structural rejuvenation. The magnitude of nutritional contribution is likely context-dependent, influenced by baseline micronutrient status, environmental exposure, metabolic health, and interaction with dermatologic treatments. Recognizing this contextual dependency prevents overstatement while acknowledging biologically meaningful modulation.

**Figure 3:**
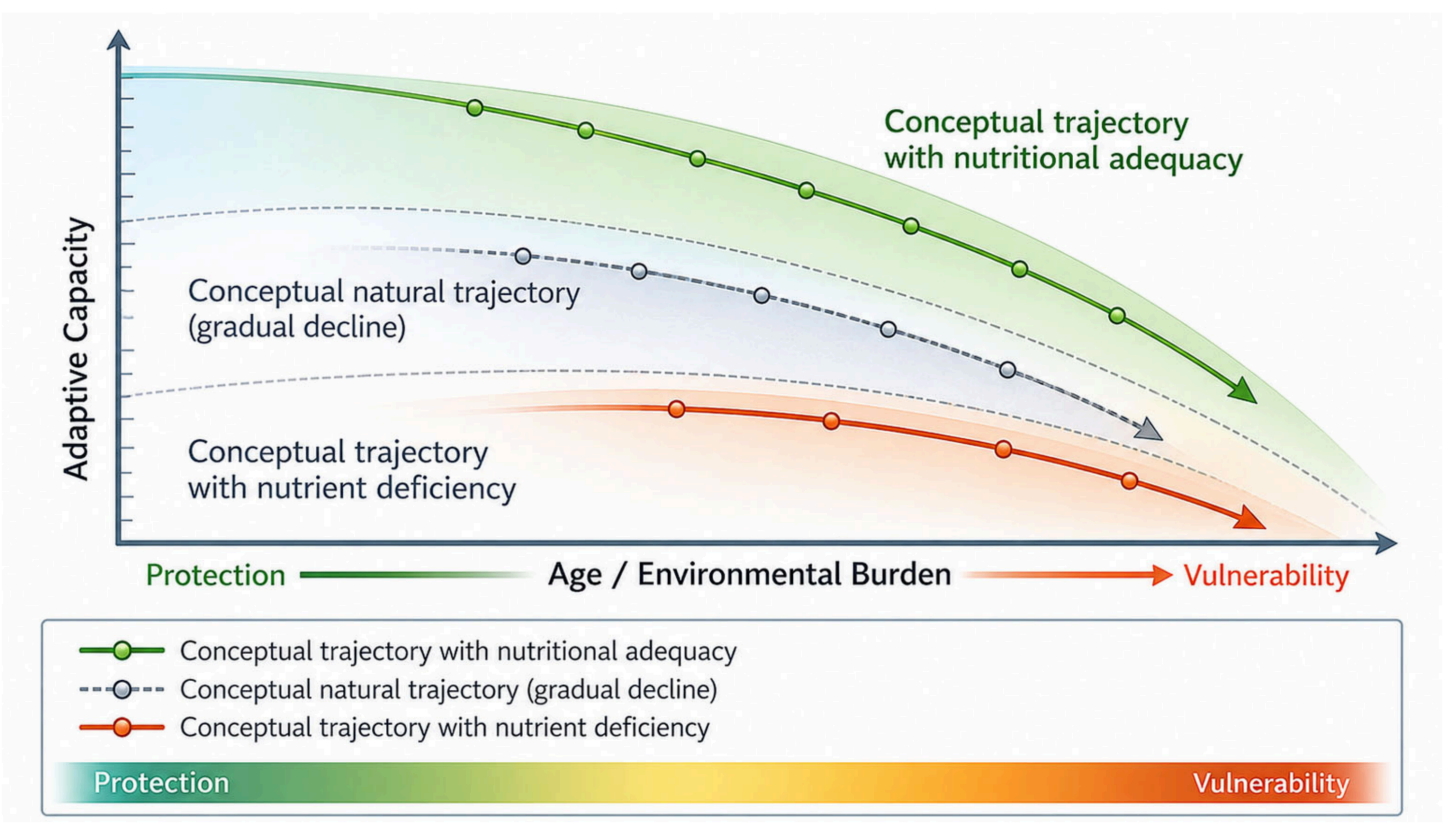
Conceptual model of skin resilience across the lifespan. The figure illustrates hypothetical trajectories of adaptive capacity under different nutritional conditions. Nutritional adequacy is presented as a potential modulatory factor that may help preserve adaptive capacity within physiological limits. This is a conceptual model based on current biological understanding; the available clinical intervention evidence is predominantly short-term and does not establish long-term modification of chronological skin aging.

The adaptive model proposed in this review is schematically illustrated in Figure 3. Rather than suggesting that nutritional interventions reverse intrinsic aging processes, the figure conceptualizes skin resilience as a dynamic adaptive range that may become progressively constrained with increasing age and cumulative environmental burden. Within this conceptual framework, nutritional adequacy is positioned as a modulatory factor that may help preserve adaptive capacity within physiological limits. Because the available clinical intervention studies are predominantly short-term, this model should not be interpreted as evidence that nutritional interventions have been demonstrated to alter long-term trajectories of chronological skin aging. Instead, it represents a conceptual synthesis in which maintenance and stabilization of cutaneous function are distinguished from structural rejuvenation.

## 10. Conclusions

Skin resilience provides a multidimensional framework through which the biological stability of aging skin can be examined across interconnected domains, including redox balance, extracellular matrix integrity, inflammatory regulation, and barrier competence. Rather than representing a novel biological mechanism, this construct integrates established dermatologic processes into an operational model that allows structured evaluation of nutritional influences. Current evidence supports a contributory role for selected micronutrients and bioactive compounds—particularly carotenoids and antioxidant vitamins—in supporting cutaneous redox stability and short-term photoprotection. Mechanistic data further suggest potential modulation of matrix remodeling pathways and inflammatory signaling. However, the magnitude and durability of clinically meaningful structural effects remain variable, with many studies limited by modest sample sizes, short intervention periods, and heterogeneous endpoints. Evidence for substantial alteration of long-term aging trajectories or pronounced structural regeneration remains insufficient.

These findings highlight the importance of distinguishing between enhancement of biochemical markers and demonstrable modification of clinically relevant outcomes. Future research should prioritize adequately powered, longer-duration randomized controlled trials employing standardized resilience-related endpoints and stratified analyses based on baseline nutritional status. Within a broader dermatologic and systemic context, micronutrients are best conceptualized as supportive modulators of adaptive capacity rather than as primary anti-aging interventions. When interpreted within this balanced framework, nutritional adequacy may contribute to the preservation of skin resilience, particularly under environmental or metabolic stress.

## Figures and Tables

**Table 1:** Controlled human intervention studies (2000–2025) investigating micronutrient and bioactive compound supplementation and measurable skin outcomes.

Study	Design/Participants	Intervention/Comparator	Route/Duration	Main Endpoints	Quantitative Results
Greul et al., 2002 [35]	RCT, double-blind, placebo-controlled; *n* = 27	Antioxidant combination vs. placebo	Oral; 8–12 wk	UV-induced erythema; MMP-1/MMP-9	No significant between-group difference for UV erythema; MMP-1 decreased vs. placebo (*p* < 0.05). Effect size/95% CI: NR.
Segger et al., 2004 [42]	RCT, double-blind, placebo-controlled; *n* = 62 women, 45–73 y	Evelle^®^ vs. placebo	Oral; 12 wk	Elasticity; roughness	Elasticity +9% vs. placebo at 6 wk (*p* = 0.0351); roughness −6% vs. control at 12 wk (*p* = 0.0157). 95% CI: NR.
Heinrich et al., 2006 [50]	RCT; *n* = 24 women	High-flavanol cocoa (326 mg/d) vs. low-flavanol cocoa (27 mg/d)	Oral; 12 wk	UV erythema; skin density/thickness; hydration; TEWL	UV erythema −15% at 6 wk and −25% at 12 wk in high-flavanol group; skin thickness 1.11→1.24 mm; TEWL 8.7→6.3 g/h·m^2^. No corresponding changes in low-flavanol group.
Kafi et al., 2007 [51]	RCT, vehicle-controlled; *n* = 36 elderly subjects	0.4% retinol vs. vehicle	Topical; 24 wk	Fine wrinkles; roughness	Fine-wrinkle score −1.64 vs. −0.08 (*p* < 0.001). Other wrinkle/roughness endpoints also favored retinol.
Heinrich et al., 2006 [52]	RCT; *n* = 39; 3 groups	Carotenoids ± vitamin E/Se vs. placebo	Oral; 12 wk	Density; thickness; roughness; scaling	Skin density and thickness increased in both active groups; roughness/scaling decreased. Exact effect sizes/95% CI: NR.
Bouilly-Gauthier et al., 2010 [49]	Controlled clinical intervention; *n* = 139 women	*L. johnsonii* + carotenoids vs. control	Oral; 10 wk	MED; UV-induced inflammatory changes	Clinical MED +20%; instrumental MED +19%. UV-associated inflammatory changes were attenuated. 95% CI: NR.
Meinke et al., 2013 [47]	RCT, double-blind, placebo-controlled; *n* = 24	Dietary carotenoids vs. placebo	Oral; 12 wk	Cutaneous carotenoids; radical-scavenging capacity	Cutaneous carotenoids increased; radical-scavenging capacity and protection against stress-induced radical formation increased vs. placebo. Effect size/95% CI: NR.
Phetcharat et al., 2015 [53]	RCT, double-blind; *n* = 34	Rose hip 3 g/d vs. astaxanthin 4 mg/d	Oral; 8 wk	Moisture; elasticity; wrinkles	Rose hip: moisture 51.55 → 62.74 (*p* < 0.05); elasticity 54.65 → 66.74 (*p* < 0.05). No significant between-group difference. 95% CI: NR.
Stephens et al., 2016 [36]	RCT, double-blind, placebo-controlled; 201 enrolled, 152 completed	Marine complex + vitamin C + zinc vs. placebo	Oral; 16 wk	Facial appearance; wrinkles; roughness; pigmentation	Several prespecified facial parameters showed significant between-group differences favoring intervention (*p* < 0.05). No significant between-group difference for TEWL/moisturization.
Laing et al., 2020 [37]	RCT, placebo-controlled, triple-blind; *n* = 60 women	Collagen peptides + micronutrients vs. placebo	Oral; 12 wk	Collagen structure	Primary endpoint showed a significant between-group difference favoring intervention; no significant improvement with placebo. Numerical effect size/95% CI: NR in the verified abstract.
Lin et al., 2021 [48]	RCT, double-blind, placebo-controlled; *n* = 50	Fish collagen + Djulis vs. placebo	Oral; 8 wk	Hydration; brightness; wrinkles; collagen	Within collagen group: hydration +17.8%, brightness +5.4%, crow’s feet +14.9%, wrinkles +29.3%, collagen content +22.3%. These are within-group changes; between-group effect size/95% CI: NR.
Xie et al., 2022 [43]	RCT, double-blind, placebo-controlled; *n* = 55	Multi-plant extract vs. placebo	Oral; 12 wk	TEWL; hydration; sebum; elasticity; pigmentation	TEWL, hydration, sebum, elasticity and pigmentation indices showed significant changes from baseline in the intervention group. Between-group effect size/95% CI: NR.
Žmitek et al., 2024 [38]	RCT, double-blind, placebo-controlled; *n* = 87 women	Collagen + vitamin C ± HA vs. placebo	Oral; 16 wk	Dermal density; texture; wrinkles	Dermal density +16.3% (CP) and +16.0% (CPHA), *p* < 0.001; wrinkle volume −13.8% and −13.9%, *p* < 0.001; maximum wrinkle depth −16.9% and −19.2%. No significant superiority of HA addition.
Rao et al., 2025 [44]	RCT, double-blind, placebo-controlled, 4-arm; *n* = 134	Oral and/or topical trans-resveratrol vs. corresponding placebo	Oral/topical; 8 wk	Wrinkles; sebum; pigmentation	A/A group had significantly lower wrinkle scores vs. P/P at week 8. Topical-active groups had significantly higher U-zone sebum vs. topical-placebo groups. Other skin parameters: no significant between-group differences.

Note: Quantitative results are reported according to the analysis presented in the original publications. Between-group differences and within-group changes from baseline are distinguished explicitly. NR, not reported. Where an original publication did not provide a numerical effect estimate or 95% confidence interval in the verified report, these values were not inferred or calculated.

## Data Availability

No new data were created or analyzed in this study. Data sharing is not applicable to this article.

## Supplementary Materials

The following supporting information can be downloaded at: https://www.mdpi.com/article/10.3390/biomedicines14092111/s1, Table S1: Database sources and search strategies used for the literature search; Table S2: Key references included in the review, categorized according to publication type, main evidence domain, and relevance to skin resilience and biological adaptability.
