# Supplementary material for: Skin Resilience and Biological Adaptability: Current Clinical Evidence on Micronutrients and Bioactive Compounds
Source: Biomedicines. 2026 Sep 18;14(9):2111. doi: 10.3390/biomedicines14092111 (PMC13604365; doi:10.3390/biomedicines14092111)
Supplement: Supplementary file 1 [file biomedicines-14-02111-s001.zip › biomedicines-4507141-supplementary.pdf]

Suppl Table S1. Database sources and search strategies used for the literature search

Database	***Search strategy***
PubMed/MEDLINE	*(("Skin Aging"[Mesh] OR "Aging"[Mesh] OR "skin aging" OR "skin ageing" OR photoaging OR "photo-ageing" OR "cutaneous aging" OR "cutaneous ageing" OR "skin resilience" OR "cutaneous resilience" OR "biological adaptability" OR "cutaneous adaptability" OR "skin homeostasis" OR "barrier function" OR "epidermal barrier") AND ("Dietary Supplements"[Mesh] OR "Diet, Food, and Nutrition"[Mesh] OR micronutrient OR vitamin OR mineral* OR "trace element*" OR carotenoid* OR beta-carotene OR lycopene OR lutein OR astaxanthin OR "vitamin C" OR ascorbate OR "vitamin E" OR tocopherol* OR selenium OR zinc OR polyphenol* OR flavonoid* OR phytochemical* OR "plant extract*" OR antioxidant* OR resveratrol OR collagen OR "collagen peptide*" OR "bioactive compound*" OR supplement* OR nutrition* OR dietary OR oral OR topical) AND ("transepidermal water loss" OR TEWL OR hydration OR elasticity OR firmness OR "dermal density" OR "collagen density" OR "collagen content" OR wrinkle* OR "wrinkle depth" OR erythema OR pigmentation OR "oxidative stress" OR "oxidative damage" OR "antioxidant capacity" OR "radical scavenging" OR "inflammatory response*" OR "inflammatory biomarker*" OR cytokine* OR photoprotection))**
Scopus	*TITLE-ABS-KEY(("skin aging" OR "skin ageing" OR photoaging OR "photo-ageing" OR "cutaneous aging" OR "cutaneous ageing" OR "skin resilience" OR "cutaneous resilience" OR "biological adaptability" OR "cutaneous adaptability" OR "skin homeostasis" OR "barrier function" OR "epidermal barrier") AND (micronutrient OR vitamin OR mineral* OR "trace element*" OR carotenoid* OR beta-carotene OR lycopene OR lutein OR astaxanthin OR "vitamin C" OR ascorbate OR "vitamin E" OR tocopherol* OR selenium OR zinc OR polyphenol* OR flavonoid* OR phytochemical* OR "plant extract*" OR antioxidant* OR resveratrol OR collagen OR "collagen peptide*" OR "bioactive compound*" OR supplement* OR nutrition* OR dietary OR oral OR topical) AND ("transepidermal water loss" OR TEWL OR hydration OR elasticity OR firmness OR "dermal density" OR "collagen density" OR "collagen content" OR wrinkle* OR "wrinkle depth" OR erythema OR pigmentation OR "oxidative stress" OR "oxidative damage" OR "antioxidant capacity" OR "radical scavenging" OR "inflammatory response*" OR "inflammatory biomarker*" OR cytokine* OR photoprotection))**
Web of Science Core Collection	*TS=(("skin aging" OR "skin ageing" OR photoaging OR "photo-ageing" OR "cutaneous aging" OR "cutaneous ageing" OR "skin*

	resilience" OR "cutaneous resilience" OR "biological adaptability" OR "cutaneous adaptability" OR "skin homeostasis" OR "barrier function" OR "epidermal barrier") AND (micronutrient OR vitamin OR mineral OR "trace element*" OR carotenoid* OR beta-carotene OR lycopene OR lutein OR astaxanthin OR "vitamin C" OR ascorbate OR "vitamin E" OR tocopherol* OR selenium OR zinc OR polyphenol* OR flavonoid* OR phytochemical* OR "plant extract*" OR antioxidant* OR resveratrol OR collagen OR "collagen peptide*" OR "bioactive compound*" OR supplement* OR nutrition* OR dietary OR oral OR topical) AND ("transepidermal water loss" OR TEWL OR hydration OR elasticity OR firmness OR "dermal density" OR "collagen density" OR "collagen content" OR wrinkle* OR "wrinkle depth" OR erythema OR pigmentation OR "oxidative stress" OR "oxidative damage" OR "antioxidant capacity" OR "radical scavenging" OR "inflammatory response*" OR "inflammatory biomarker*" OR cytokine* OR photoprotection))**

Suppl Table S2. Key references included in the review, categorized according to publication type, main evidence domain, and relevance to skin resilience and biological adaptability

No.	*Reference*	*Publication type*	*Main topic / evidence domain*	*Relevance to the present review*
1	*Baker et al., 2023*	*Narrative review*	*Skin barrier function*	*Barrier integrity and cutaneous homeostasis*
2	*Rittié & Fisher, 2015*	*Review*	*Natural and sun-induced skin aging*	*Biological basis of intrinsic and extrinsic aging*
3	*Kammeyer & Luiten, 2015*	*Review*	*Oxidation events and skin aging*	*Oxidative stress and aging*
4	*Masaki, 2010*	*Review*	*Antioxidants in skin*	*Antioxidant mechanisms and anti-aging effects*
5	*Alexander et al., 2018*	*Methodological review*	*Transepidermal water loss*	*Assessment of epidermal barrier function*
6	*Hughes et al., 2021*	*Longitudinal human study*	*Dietary antioxidant capacity and photoaging*	*Relationship between nutritional antioxidant status and skin aging*
7	*Korkina & Pastore, 2009*	*Review*	*Redox regulation in skin*	*Redox homeostasis and inflammatory skin processes*
8	*Battie et al., 2014*	*Review*	*UVA-induced damage and photoaging*	*Environmental stress and photoaging*
9	*Lan et al., 2019*	*Experimental human study*	*UVA-induced skin aging*	*UV-related structural and functional changes*

10	*Pilkington et al., 2021*	*Review*	*Inflammaging and skin*	*Chronic inflammatory processes in aging skin*
11	*Lee et al., 2021*	*Review*	*Cellular senescence and inflammaging*	*Senescence-associated inflammatory signaling*
12	*Chin et al., 2023*	*Review*	*Cellular senescence in skin aging*	*Cellular mechanisms of cutaneous aging*
13	*Stanescu et al., 2025*	*Systematic review*	*Carotenoids and skin aging*	*Clinical and mechanistic evidence for carotenoids*
14	*Bjørklund et al., 2025*	*Review*	*Trace elements and skin health*	*Nutritional role of trace elements*
15	*Ho & Dreesen, 2021*	*Review*	*Cellular senescence in skin*	*Senescence-related mechanisms*
16	*Marionnet et al., 2014*	*Review*	*Solar UV exposure and photoprotection*	*Environmental stress and photobiologic responses*
17	*Fluhr et al., 2024*	*Narrative review*	*Epidermal barrier function*	*TEWL, hydration and barrier competence*
18	*Rousselle et al., 2019*	*Review*	*Extracellular matrix and skin repair*	*ECM organization and tissue integrity*
19	*Feng et al., 2024*	*Review*	*Matrix metalloproteinases and photoaging*	*ECM degradation and photoaging*
20	*Quan et al., 2009*	*Review*	*Matrix-degrading metalloproteinases*	*Collagen degradation and photoaging*
21	*Zhang et al., 2024*	*Review*	*Dermal fibroblast senescence*	*Dermal aging and fibroblast dysfunction*
22	*Shin et al., 2019*	*Review*	*Molecular mechanisms of dermal aging*	*Oxidative stress, ECM remodeling and aging*
23	*Chen et al., 2021*	*Review*	*Oxidative stress in skin*	*Redox imbalance and cutaneous protection*
24	*Piipponen et al., 2020*	*Review*	*Keratinocyte immune functions*	*Cutaneous immune regulation and adaptive responses*
25	*Sreedhar et al., 2020*	*Review*	*Mitochondria in skin health and aging*	*Mitochondrial function and systemic context*
26	*Baswan et al., 2021*	*Review*	*Ingestible carotenoids*	*Clinical evidence for carotenoid-mediated photoprotection*
27	*Heinrich et al., 2003*	*Controlled human study*	*β-Carotene/mixed carotenoids and UV erythema*	*Human photoprotection evidence*
28	*Groten et al., 2019*	*Randomized controlled trial*	*Tomato phytonutrients and UV response*	*Photobiologic response to nutritional intervention*
29	*Pullar et al., 2017*	*Review*	*Vitamin C and skin health*	*Collagen synthesis and antioxidant function*

30	*Pincemail & Meziane, 2022*	*Review*	*Vitamin E/selenium*	*Oral antioxidant supplementation and skin health*
31	*Keen & Hassan, 2016*	*Review*	*Vitamin E in dermatology*	*Antioxidant and dermatologic applications*
32	*Natarelli et al., 2025*	*Systematic review*	*Oral supplements and photoprotection*	*Clinical evidence for oral nutritional interventions*
33	*Cole et al., 2018*	*Review*	*ECM regulation and fibroblast function*	*Structural mechanisms of skin aging*
34	*Grether-Beck et al., 2017*	*Double-blind placebo-controlled crossover study*	*Lycopene/lutein and UV protection*	*Molecular evidence of oral carotenoid photoprotection*
35	*Singh et al., 2025*	*Comprehensive review*	*Dietary phytochemicals and premature skin aging*	*Nutritional modulation of skin-aging pathways*
